# Differences in Health: The Influence of Gender and Institutional Settings on Sickness Claims in Gothenburg, Sweden (1898–1950)

**DOI:** 10.1093/shm/hkz019

**Published:** 2019-04-09

**Authors:** Helene Castenbrandt, Barbara Ana Revuelta-Eugercios, Kjell Torén

**Affiliations:** 1 Saxo Institute, University of Copenhagen, Karen Blixens Vej 4, 2300, Copenhagen, Denmark; 2 Section of Occupational and Environmental Medicine, Institute of Medicine, University of Gothenburg, Box 414, 405 30 Gothenburg, Sweden

**Keywords:** sickness funds, friendly societies, morbidity, gender, sickness claims

## Abstract

Sickness funds information has given conflicting evidence on the evolution of morbidity during the mortality decline. Evidence on increased morbidity has been explained by an actual increase of morbidity, a cultural inflation of morbidity or changing institutional settings, however, morbidity rates have also been shown to be stable over time when age composition of members is controlled for. Most previous studies have been confined to data on men; however, in an earlier article, Castenbrandt found large gender differences in historical sick leave by using national statistics on both men and women. To move forward, this article aims to analyse trends in sickness claims during the mortality decline in the early twentieth century using individual level data from Swedish sickness funds covering the period 1898–1950. Concretely, we investigate gender differences in sickness claims (incidence and duration) and how institutional settings (member composition and fund-specific regulations) affected the sick leave patterns.

Ester, aged 23 and married, was registered in *Sömmerskornas Sjuk- och Begravningskassa* (SSBK), a sickness fund for seamstresses in Gothenburg, Sweden, in 1909 and remained a member for the rest of her life.^1^ She claimed maternity benefit for the eligible 14 days on two occasions, but she claimed no sickness benefit until she reached the age of 53 years; she then suffered the first of three long-term sickness episodes leading up to her death 3 years later. During Ester’s lifetime, life expectancy in Sweden rose by 15 years. It is, however, unclear whether these additional years her children’s generation got to live were spent in good health or were plagued with disease.

Information on sickness histories, such as Ester’s and fellow members of sickness funds, has been used by several researchers in trying to entangle the relationship between mortality and morbidity. Riley has argued that morbidity rose as mortality declined, while Harris and Gorsky *et al*. have underlined larger patterns of stability in morbidity from the late nineteenth century into the mid-twentieth century.^2^ Some scholars, however, remain unconvinced as to whether or not we can infer morbidity from these types of sources.^3^ For example, there are indications that the voluntary funds’ selection criteria, keeping high-risk individuals out, lowered the overall level of sickness claims compared to company-based compulsory funds.^4^

Most of these studies have been based on male accounts as sickness insurance was tied to wage-earning, and much of the available information refers only to men. However, women were also wage earners, and by 1940, there were as many female members in Swedish sickness funds as there were male members. Adding women’s health to these accounts is particularly important as historical mortality differences, contemporary health research, as well as historical accounts on health, all point to the existence of large health differences by sex, although the direction is somewhat unclear.

Contemporaries considered women frailer than men and excluded them from many sickness funds as they were deemed ‘high risk’ and likely to increase the funds’ expenditure.^5^ However, Castenbrandt’s analyses of Swedish national published statistics for the period 1900–50 has shown that men tended to have more frequent sickness episodes, although shorter, when compared with women. After law changes in the early 1930s, the differences in incidence almost disappeared while women’s more prolonged sickness duration became more apparent. However, the limitations of this aggregated data did not allow for a deeper analysis of sickness claims in relation to institutional settings such as member composition and fund-specific regulations.^6^

This article will study changes in morbidity by taking advantage of data from sickness funds, using the sickness histories of thousands of men and women that were members in two sickness funds, one male-based and one female-based, for the period 1898–1950. We investigate gender differences in sickness claims (incidence and duration) and how institutional settings (member composition and fund-specific regulations) affected sick leave patterns.

The section ‘Analysing Sickness in the Past’ sets out the main theses and empirical evidence on trends of morbidity and sickness claims in Europe and shows the importance of the article’s focus on gender differences and institutional settings. The section ‘Institutional Settings: The Effect of Legislation and Fund-specific Regulations’ gives an overall contextualisation to sickness funds in Sweden and presents the two funds. The section ‘The Gothenburg Sickness Funds Data set’ discusses the data set and method, along with an analysis over the member composition of the two funds. The section ‘Institutional Settings and Gender Differences’ examines trends in incidence and duration of sickness claims while discussing the influence of institutional settings and gender. The last section provides a brief conclusion and discussion of the results.

## Analysing Sickness in the Past

With increasing longevity and rising costs in the health sector, the relationship between morbidity and mortality trends has come into spotlight. Gains in life expectancy are not necessarily gains in healthy years; instead, present-day statistics suggest that we live longer but sicker lives. The historical community has for decades tried to answer if this is a widespread phenomenon in the historical health transition or if it is a recent development, related to gains at later ages.

While deaths are relatively easy to capture in historical records, health is a much more difficult outcome to gather historical data about. There is a long tradition of assessing health from anthropometric measurements such as height and weight to gain indirect measures of health. But these measures can only be an indication of health and disease in childhood and cannot explicitly account for the role of sickness among the adult population. In parallel, the level of morbidity in a society has been investigated through the records of friendly societies and sickness funds. This was the approach undertaken by Riley in the 1980s and 1990s. In a series of publications, he argued that the evidence from the *Ancient Order of Foresters* showed that morbidity, as measured by the duration of sickness spells, rose between 1880 and 1914, while mortality rates fell.^7^ This finding sparked a controversy, where his sources, methodological approach and interpretation were all called into question. In contrast, Murray cast doubt over the relationship between sick leave and morbidity, instead arguing that trends in sickness claims were an effect of institutional settings related to economic and political constraints of the sickness funds. According to him, voluntary funds, in comparison to compulsory company-based ones, had to cope with adverse selection (tendency of the individuals with poorer health to seek membership into a fund), which jeopardised the financial stability of the fund and may have reduced the willingness of the fund to pay sickness claims.^8^ Sheila Ryan Johansson, on the other hand, accepts the connection between sick leave and morbidity but challenges the meaning of the rising number of sick days, explaining it through a ‘cultural inflation of morbidity’. She claims that a change in perception and attitudes towards disease and its reporting occurred, emphasising that sickness reporting is not only a biological but also a culturally constructed phenomenon. Meaning that the rise in reported sickness followed in response to a lowered threshold for reporting, while actual morbidity remained the same.^9^

Other researchers, however, have accepted both the sources and the interpretation, but focused on the limitations of Riley’s methodological approach. By using more detailed material, moving away from aggregated statistics and instead relying on individual sickness histories from members of the *Hampshire Friendly Society*, Harris and Gorsky *et al.* were able to gauge the effect of age distribution, length of stay, membership behaviour and types of diseases, thus overcoming some of the limitations of Riley’s material. Their results indicate a similar crude increase in general morbidity rates following Riley’s approach. However, a detailed age-specific analysis, which took into account the changing member composition, revealed that the general rise in sickness incidence over time corresponded to an increasing proportion of older members, who in general made more claims. Their analyses do not provide support for either increased morbidity or the cultural inflation of morbidity.^10^

This debate has mainly concerned British localities. Among the exceptions are the previously mentioned work of Murray, who used aggregated data from several countries, and a recent number of publications on Sweden. Andersson and Eriksson have used information on policy and aggregated statistics concerning 847 societies in Sweden covering the period 1900–10 to analyse differences between voluntary and compulsory funds. Compulsory funds tended to actively decrease duration, while voluntary sickness funds generally accepted sickness spells with longer duration. Voluntary funds did, on the other hand, report fewer sickness claims. Anderson and Eriksson suggest that the higher social control, and longer waiting periods, in voluntary funds reduced moral hazard and brought down the number of short-term sickness spells.^11^ With a wider perspective, Castenbrandt analysed Swedish official statistics from 1900 until the private funds came under state regulation in 1955. Her results show an increasing number of sickness cases, as well as increasing number of sick days per member over time.^12^ However, given that the published statistics do not provide information about member composition, it is still unclear to what extent these changes represent increased morbidity or reflect the legislative changes.

Castenbrandt’s study also introduced data on women, which showed very interesting gender differences. Women reported a lower number of sickness cases but a higher number of sick days per sickness case, meaning fewer but longer episodes than men. This evidence the contemporary notion on frailer women that were expected to be sick more frequently than men. In addition, also, Andersson and Eriksson have found that societies that accepted both men and women on average had fewer sickness incidences than female-excluding funds, supporting the evidence of fewer sickness cases for women.^13^

Interestingly, as female life expectancy was higher for women than for men (2.5 years^14^) also in early-twentieth-century Sweden, these findings do not clearly fall into the female health paradox found in contemporary societies, which shows women to have a higher life expectancy while using more health services, reporting worse self-rated health, and having higher morbidity.^15^

Present-day morbidity differences between men and women are often viewed through the distribution of various diseases. Men are shown to have a higher prevalence of more life-threatening conditions, such as cardiovascular diseases, than women. In contrast, chronic and debilitating disorders and damaging but less life-threatening diseases, such as anaemia, autoimmune diseases and rheumatoid disorders, are more commonly reported among women. The effect is that men more often die from their conditions, even though women report more illness than men.^16^ Not only biological risk factors have been put forward, but also different risk of exposure to infections and social explanations such as the division of labour within families, differential composition of the male and female workforce, and reluctance among male subjects to seek health care.^17^

Moreover, as stated earlier, the connection between sickness and sickness claim could be biased by cultural norms on reporting, so it is relevant to also consider the literature that emphasises gender inequalities at home and at work, as well as cultural aspects related to the perception of disease and its reporting. Studies using contemporary data have found that responsibilities related to parenthood had a high effect on sickness rates. Young mothers in particular had high absenteeism, while men with children had fewer sickness spells than those without children.^18^ Here the difference between sickness and absence is highlighted as gender roles, that have given women higher responsibilities for children and placed men as breadwinners, likely contribute to creating divergent patterns of absenteeism regardless of actual sickness. Furthermore, there is evidence supporting the thesis that gender differences in sickness absenteeism have been culturally constructed by heavily gendered norms. Eric Patton and Gary Johns have analysed articles from the *New York Times* on women’s absenteeism in the time period 1851–2004.^19^ They found that since WWII, women’s absence is most commonly described as derived from difficulties in balancing work with childcare and other family duties. Men’s absence, on the other hand, is more often associated with deviant behaviour, such as unsanctioned absence and conflict issues. In that sense, men’s absence is portrayed as more of a problem than women’s, potentially making women’s absence more socially acceptable than men’s.^20^

Only through the analysis of detailed information on sickness histories can we start to untangle the gender differences and effects institutional settings have had on trends in sickness claims. Thus, this article will investigate how the Swedish experience, as told through detailed sickness histories, can help us understand the mechanisms of sick leave and its relationship to morbidity.

## Institutional Settings: The Effect of Legislation and Fund-specific Regulations

The expansion of associations to provide mutual support in case of sickness took off in Sweden, as in many other European countries, as industrialisation and urbanisation in the nineteenth century changed the lives of the working population. While these funds became compulsory in places like Germany, in Sweden, they remained voluntary.^21^ However, they were slowly placed under state regulation during the first half of the twentieth century with legislations passed in 1892, 1910 and 1931, gradually making the funds more homogeneous. The law of 1892 was not very detailed, but it was the first step in bringing the funds under state supervision as it required the funds to be registered in order to receive state subsidies. The greatest change happened when the 1931 law was implemented in 1934, when a reorganisation of funds strengthened the role of central sickness funds overseeing the local funds, ensured more uniform rules and opened up all funds to both men and women.^22^

The City of Gothenburg was founded in the early seventeenth century. The city was built around trade, with extensive foreign exchange. Even so, the textile industry was an important employer for the city’s workers already in the mid-eighteenth century.^23^ Being the second largest city in Sweden, Gothenburg developed as a modern industrial city by the late nineteenth century, experiencing a substantial population growth, from about 100,000 people in 1890 to just over 350,000 in 1950.^24^ This development was led by the expansion of the textile industry; however, the city became more and more dominated by shipyard and metal factories during the twentieth century. With the city’s growth, its labour market also expanded into the service sector side.^25^

In 1901, the 128 registered sickness funds in Gothenburg had around 12,000 members, and only 20 of these funds admitted women. Membership numbers had increased to over 160,000 by 1950, and in the same time, the proportion of female members increased from under 10 to over 50 per cent.^26^ These changes were consistent with the national development.^27^ The expanding sickness fund movement played an important role in bettering the economic conditions for the growing urban working class. In general, the Swedish working class had to rely on poor relief in times of need. The municipalities were responsible for such aid, which often were consistent with placement at workhouses, and later, homes for the elderly. The organisation of poor relief and health care transformed in Sweden during the first half of the twentieth century; however, receiving such aid generally required that you had no other means.^28^ Therefore, the growth of the sickness fund movement was part of a development where more and more working class groups found themselves above the poverty line and in need of new solutions in time of temporary economic difficulties.

SSBK and Redbar were two of the newly established voluntary funds in the city of Gothenburg in late nineteenth-century Sweden. They were for women and men, respectively, and they shared many traits that characterised voluntary association from early on. They both started out as small funds and existed for a long period of time and had stable organisations, which makes them very suitable for a study of long-term trends.

Ester’s fund, SSBK, started as a sickness and funeral fund restricted to seamstresses in response to what was considered their tough working conditions. There was great interest, and 60 seamstresses attended the first meeting in February 1898.^29^ Statutes from 1914 stated that the fund was open to all seamstresses living in Gothenburg and neighbouring municipalities. However, after a rule change in 1923, all women, not just seamstresses, were entitled to membership, although the fund kept its old name, SSBK (*Sömmerskornas Sjuk- och Begravningskassa;* Seamstresses Sickness and Funeral Fund). As a result, shop assistants, clerks, maids, dental assistants, etc. started to be accepted as members. However, the fund was restricted to women until the 1931 legislation was implemented in 1934, which opened admission to men and instituted a change of name to Tryggheten. By 1946, 10 per cent of members were men.^30^

The second fund, Redbar, was probably founded around 1884, as that is the earliest admission year we have found. However, records from its earliest years are missing, and surviving sickness records start at 1890. Just as SSBK, Redbar had restrictions influencing the member composition. For many years, it only accepted men with a respectable reputation, who came with a recommendation from an existing member, and that were in good health. But from 1934, no recommendation was needed, and women could also become members. As a result, by 1945, over 40 per cent of members were women.^31^ As Redbar was not restricted to a single working group, it attracted members from different occupations, such as factory workers, warehouse workers, carpenters, drivers, shop assistants, and after 1934, female work groups such as maids and seamstresses.

Besides these strict criteria for admission, compliance with the regulation was another means of ensuring social control. Members had to comply with the rules while sick. Moreover, members had to personally pay the membership fee at monthly meetings, which ensured that members were not estranged from one another but instead met and socialised. In fact, practices that hindered this personal contact with the fund were for long discouraged. For example, the board of Redbar refused to open up a post office account in 1930, which had been suggested so that members would not have to go to meetings for the payment of membership fees. However, the board found it important that members had to personally attend the meetings for this reason.^32^

In addition, members were required to actively participate as sick visitors, checking on other members receiving benefits. The chairperson distributed the turns and visits among members, for which members received no compensation, but could be fined if not properly performed. However, with time this became too burdensome, and the question of paid sick visitors was discussed during several of SSBK’s board meetings in 1931. Apparently, many members would rather be fined than do their sick visits, which had led to the temporary solution that the chairperson herself did many of the sick visits for a small amount of compensation.^33^ Soon after, both funds instituted permanent paid sick visitors.

The sick visitors were in charge of supervising members’ compliance with the strict regulations prescribed for sickness periods. For instance, sickness had to be reported to the chairperson in due time. The member then had to receive sick visitors during the illness and follow the doctor’s instructions, thereby doing everything considered necessary to regain good health.^34^ Failure to do so could lead to suspension of payments, as in the following example reported in the board minutes of SSBK in 1911. The problem started when the sick visitors could not find the member at home, which prompted the chairperson to pay her another visit late one evening. As she was once again found to not be at home, the chairperson called the doctor, who explained that the patient had been ordered to only go outside in nice weather during the middle of the day. The fact that the member receiving sick pay had not complied with the statutes by not receiving the sick visitors, and more so that she went against the doctor’s instructions by being away late in the evening, led the board members to suspend her sick pay.^35^

Over time, the local sickness funds transformed from general benevolent funds to handling only sick pay. Initially, members could get compensation for funeral expenses, medication and hospital care, sometimes even covering family members, as well as paid sick leave.^36^ From 1908, SSBK also covered 14 days of maternity leave and following the occupational safety laws from 1912, some additional days if the mother was deemed unfit for work.^37^ However, with the changes in 1934, the funeral coverage was transferred to a separate fund. Also, the central fund, *Göteborgs stads erkända centralsjukkassa* (GCSK), now administered hospital care, maternity benefits and benefits for doctor visits and medicine, as well as long-term sick leave. With this reorganisation, Redbar and SSBK became 2 of 14 local sickness funds in Gothenburg. Some years later, starting in 1946, all local funds in Gothenburg were successively incorporated directly into GCSK, with SSBK’s members being transferred in 1946 and Rebar’s members in 1950.

In both Redbar and SSBK, sick pay was paid after the first 3 days of illness, which was much more favourable than for the average in the country for other voluntary societies, which was around 7 days. On par with the national average, the maximum number of days for the same sickness claim over the whole period remained 90. Although, the precise regulations concerning what happened after the 90 days changed over time. Before 1933, members could be granted sick pay for a maximum of 90 days in a 12-month period. In addition, SSBK specified a total limit of 180 days for the same sickness and 270 days in total, while Redbar specified that a member could get a maximum of 270 days for the same illness and thereafter was only entitled to funeral grants.^38^ However, the statutes from 1933 gave exactly the same rights in both funds, stating that a member could claim a maximum of 90 days for the same sickness case. If a new sickness case occurred within 90 days from a previous case, it would be counted as the same case. After these 90 days, the member would be transferred to GCSK, which in this new system handled all long-term illnesses.^39^

Requirements of admission, and the tight control imposed on members, show the importance of fund-specific regulations, especially before the implementation of the 1931 legislation. We can assume that these regulations would have had an effect on the composition of members and also on their behaviour, which will be demonstrated in the following section. However, these regulations have also left us with a wealth of information on these funds in the form of personal records of members, minutes from meetings of the board and the economics surrounding it, allowing us to reconstruct individual sickness histories. The statistical analyses of this article are based on the digitisation of these sickness histories for all members of SSBK and Redbar during the periods 1898 and 1950.

## The Gothenburg Sickness Funds Data Set

In order to keep track of the members and their sickness cases, the funds carefully registered every episode in the records (dates, diagnoses and additional notes), which offer a great opportunity to study sick leave. We can reconstruct detailed sickness histories using the exact number of days and dates on which members claimed benefits, allowing us to overcome some of the limitations of previous studies that have not been able to separate incidences within the same year or quarter, making it difficult to analyse sickness duration.

In the construction of the GSF (Gothenburg Sickness Funds) data set, great care has been taken so that the full sickness history of each member is maintained, which was especially problematic as registration systems changed over time. The earliest records from SSBK comprise annual lists of members, with added information on membership payments and sickness compensation. However, in 1914, they changed to a system of ledgers similar to the one that Redbar used from the start, where a double page of the ledger was used for each member, stating personal details at the top and with plenty of space below for listing information on each member’s sickness claims. From 1934, both funds, complying with the new legislation, changed to a system of individual cards that displayed the membership data on the front side, while information on sickness claims was continuously added on the back. All members were re-registered as a new registration system was implemented but without carrying through information on their previous sickness history. Thus, for the GFS data set, individuals have had to be identified across the different registration systems to create complete sickness histories. Also, membership number proved not to be a unique identifier. The funds frequently reused numbers, so identification have been done using membership numbers in combination with personal details. Moreover, with the change to a card system in 1934, Redbar assigned all members new membership numbers, and everyone, even those members already existing in the fund, was registered with admission date 1 January 1934, so the date of admission in the GFS data set has been retrieved from the earlier ledgers, making sure that the original entry date was kept.

However, while aggregated together for analytical purposes, these circumstances make both the material and the phenomenon that it captures a bit heterogeneous, responding to both legal changes and organisational changes, which are also reflected in their size, especially after 1930.

The sources have yielded 7,535 records with membership information, from which 3,708 unique individuals have been identified (1,685 from SSBK and 2,023 from Redbar) linked to 12,197 records on sickness and compensation claims, here referred to as the *GSF data* *set*. In addition to information on membership and sickness history, the records help us get a glimpse of the members, with information such as name, sex, date of birth, occupation and place of residence.

While we know very accurately when members entered the funds, the records in some cases are less informative about when they left the fund, whether due to death, transfer or any other reason. Thus, the *GSF data* *set* has been supplemented with institutional records, where the board discussed the operational aspects of the fund, as well as the annual statistics the funds sent to the authorities (including lists of exits, transfers and deaths).^40^ This has allowed us to limit the number of members without a reported exit date to 405 individuals (10.92 per cent). However, for most of these, we can at least estimate a reliable last day alive in the fund to offer a conservative estimation of length of inclusion and thus exposure to risk of sickness. There are only 141 members that have no exit date and show no additional record after their admission.^41^ Such members had to be excluded from our analysis but, as numbers are very small and equally distributed over time, it does not pose a problem to the analysis of changes over time. The final number of members we analyse is 3,567.

For the analysis, some of the 12,197 claims in the GSF Data set have been excluded. Such claims include payment of funeral expenses for the member or relatives, information on sickness history prior to entry into the fund (available for some members transferring from other funds) as well as the maternal benefits SSBK gave women after childbirth. Moreover, sickness cases that were administratively split into two episodes, as the sickness period lasted over a new year, the first ending in 31 of December and the second starting in 1 of January, have been reconstructed so that these cases are recorded as one single claim.^42^ Also, eight cases with incomplete dates have been excluded, as well as those experienced by members omitted from the analysis, leaving the GSF data set with 11,012 unique sickness cases. These cases pertain to 2,636 members of the full 3,567 members with exit dates, meaning that 26 per cent of all members never received any sickness benefit. Moreover, a little more than 50 per cent of the members had between one and five sickness episodes, and it was unusual for members to make many sickness claims. Here the two members in Redbar that each received benefit for as many as 29 different sickness episodes were clear exceptions.

It is important to note that military service could have had an effect across this period. Soldiers were exempted from payment of membership fees while in military service and consequently could not claim sickness benefit from their local fund during such times. However, such information was not stated in the membership records. So, it is possible that we are underestimating the number of sickness cases among men, especially during the two world wars.

**Fig. 1:**
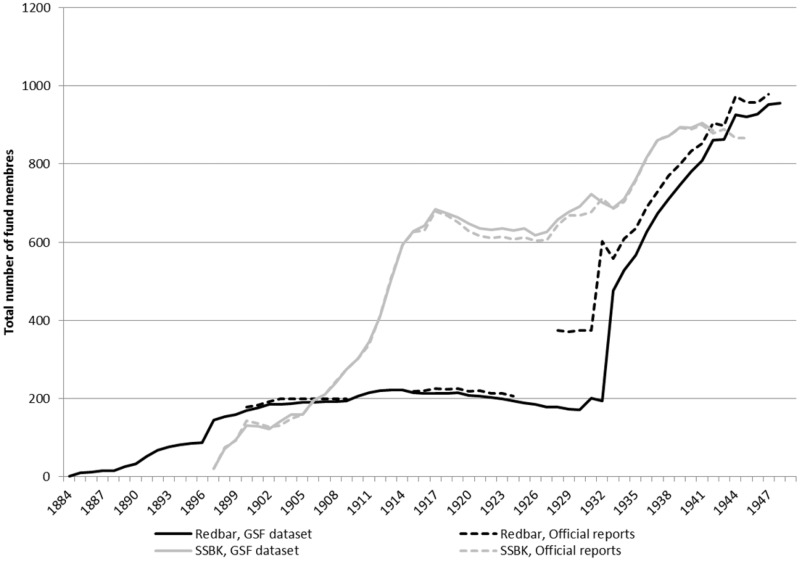
The number of members in the GSF data set and according to the funds’ official reports. The graph excludes individuals without an end date, reducing the sample to 1908 for Redbar and 1659 for SSBK. *Source*: GSF data set, and official reports: Landsarkivet i Göteborg (GLA): *Sömmerskornas sjuk- och begravningskassa (SSBK)* and *Sjuk- och begravningskassan Redbar*.

To test the accuracy of the membership inclusion in the GSF data set, Figure 1 compares the number of members according to the funds’ yearly statistics and our own computation using the inputted inclusion periods. The graph displays a close match between the statistics and the GSF data set. However, a larger dissimilarity can be seen in Redbar between 1926 and 1932, where the official reports have much higher membership numbers than our calculations from the GSF data set. This is most likely due to that the fund started to report the total membership following a merger with two other funds before they finally merged in 1933, while the archival material only gives information from the two added funds after the new system of registration was implemented in 1934.

The size of the two funds was similar at the start and end of our time period, but their trajectories were substantially different. As Figure 1 indicates, both funds had relatively low membership rates in the early years (reaching about 200 in 1906) and ended with almost the same number of members in the 1940s (around 900). During its first 20 years, SSBK had a continuous increase in members, reaching almost 700 members in 1918, followed by a 15-year period of stagnant or even declining membership rates. However, by 1934, the number of members started to increase again. Redbar, on the other hand, kept its membership to around 200 members for a long time and then increased the number of members radically when it merged with two other smaller sickness funds in 1933.

**Fig. 2:**
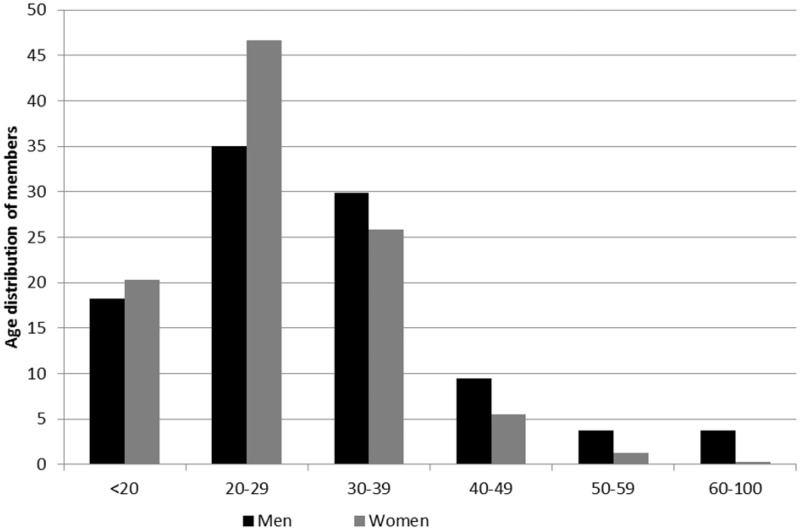
Age at time of joining the funds for men and women, 1884–1950. *Source*: GSF data set.

In relation to age composition, most members were under 40 years of age when they joined the funds. More precisely, many were in their 20s when they enrolled (see Figure 2), particularly in the case of women, where we see that almost half of admissions were in that range. Even so, the statutes from both funds posed the same age limitations for membership. In 1914, the statutes stated that new members should be aged over 18 years, but not over 45 years.^43^ The statutes from 1934 lowered the entry age to the range between 15 and 40 years.^44^ Nevertheless, some members were over 50 years when they for the first time were registered in Redbar, but these were in fact previous members of the two sickness funds that merged with the fund in 1933 and not actual new members.

Even though most members were loyal members that stayed on in the funds for many years, the fluctuations in membership recruitment affected the average length of inclusion in the funds. Many of the members that were transferred into Redbar in 1933 stayed until the fund merged into GCSK in 1950. These members with exactly 16 years in the fund constitute 6 per cent of all individuals in the data. Also, around 20 per cent of the members were recruited after 1940, which means that those members have fewer than 10 years in the fund. Moreover, around 20 per cent were members for 20 years or more, and around 10 per cent were members for less than a year.

The membership details generally included information about occupation, although it was not always stated. Members did, however, need to earn wages to be part of the funds and to be eligible to claim sickness benefit. It was not until the law from 1931 that individuals could be member of a sickness fund even if they did not have a job. But, in such cases, the member would not have the right to claim sickness benefit but only the right to compensation for the cost of health care.^45^ Employment was needed for membership in both Redbar and SSBK, and also, all members’ reports cards state that they had the right to claim sickness benefit, so we can assume that both male and female members were working even though an occupation was not always stated.

In general, the men who entered into Redbar, and from 1934 into SSBK, came from the middle to lower part of the social class distribution. They can mainly be classified as foremen, medium skilled and to a large proportion unskilled, as we can see from Table 1. However, over time, the occupational distribution shifted upwards and spread out, with the proportion of unskilled workers decreasing from 55 per cent to 13.8 per cent. The more common occupations among men were caretaker/utility man and factory worker.

**Table 1.** Social class of members according to period of entry, 1884–1950

	1884–1891	1892–1900	1901–1909	1910–1920	1921–1933	1934–1940	1941–1950
Men							
Only reported with family relation	0	2	2	9	1	0	1
Students	0	0	0	0	3	2	11
Higher management and professionals	0	0	0	1	0	1	6
Lower professionals and clerks	8	12	9	16	21	17	20
Foremen and medium skilled	30	23	30	32	22	27	20
Low skilled	5	17	6	8	32	23	22
Unskilled	55	46	51	31	19	26	14
Undefined	0	0	0	1	0	2	1
Missing	3	0	2	2	2	3	4
Total	100	100	100	100	100	100	100
Women							
Only reported with family relation		0	0	1	6	24	32
Students		0	0	0	0	1	7
Higher management and professionals		0	0	0	0	1	1
Lower professionals and clerks		0	0	0	3	21	18
Foremen and medium skilled		0	0	0	0	2	2
Low skilled		100	100	99	12	19	13
Unskilled		0	0	0	1	2	2
Undefined		0	0	0	2	13	16
Missing		0	0	0	75	19	9
Total		100	100	100	100	100	100

*Note:* Occupations have been coded through Hisco and classified by the Hisclass scheme, which has been further reduced to these seven categories. Women from SSBK entering before 1923 have been coded as low skilled, even if occupation was not stated, in accordance with the fund’s policies.

*Source*: GSF data set.

More women than men were listed without an occupation, with a description of their position in their family often stated instead (such as wife or mother). Reporting an occupation was unnecessary in SSBK until 1923, as the regulations stated that only seamstresses were accepted. However, the practice of leaving the occupation blank continued in many cases even after 1923. In Redbar, which included women after 1934, women were often reported with a family relation instead of an occupation, and more than 40 per cent had no reported occupation or just a family relationship. This practice would suggest that the actual work they carried out was not as important as for men, or not a defining attribute, which is a common issue in the analysis of women’s work and economic activity. From the limited information on occupations that was reported in the two last periods, however, we can see that the social class extraction of women who entered the fund also widened. Thus, we can track the gradual extension of the catchment area of sickness funds, with both female and male members entering them from a much more diversified set of occupations. Besides seamstresses and other similar tailoring-related activities, the more common occupations for females were shop assistant and maid.

The widening and more diversified distribution of members in both funds during the first half of the twentieth century also reflected the changing occupational structure in the city of Gothenburg. From its great expansion as an industrial city up until the 1920s, the town gradually became more diversified with a rapidly growing service sector.^46^

## Institutional Settings and Gender Differences

**Fig. 3:**
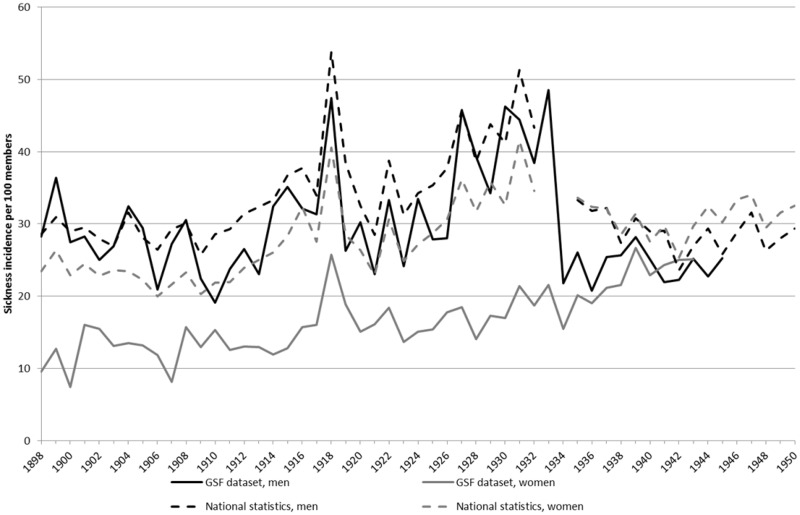
Sickness incidence per 100 members in the national statistics and the GSF data set, 1898–1950 (yearly average). *Source*: GSF data set; Castenbrandt, ‘Trends in Morbidity’.

The GSF data set follows the same increasing trend in the incidence of sickness claims over the period 1900–50 as in the national statistics, as well as documenting a similar pattern of sex differences, i.e. a higher number of sickness claims for male members (see Figure 3). However, the gender differences are much more striking in the GSF data set. Males in the GSF data set had about the same number of sickness cases and followed the same trend as in the national statistics, i.e. slightly upwards (but very volatile) until 1934, followed by a drop to a stable period afterwards. The level was slightly lower than the national statistics, but the difference was marginal.

The women in the GSF data set tell a somewhat different story. Their number of sickness cases was half of the national average between 1898 and 1934 (when all females belonged to SSBK), with sickness cases per 100 members ranging from around 10 to 20 per year. Only during the influenza epidemic in 1918 did that number rise to 25; however, there was still no overlap with either the male figures in the GSF data set or the national female figures. Only during the last period, 1935–50, did men and women in both the national statistics and the GSF data set achieve the same average number of sickness cases.

While a real health advantage for the women could theoretically be a possibility, it is likely that these substantial differences were the result of fund-specific regulations, as the funds were gender segregated until 1934, which influenced both decision-making and the culture regarding sickness claims. However, they could also be related to substantial differences in the age composition of the members across the different funds and over time.

Thus, we calculate the average sickness rates for four different age groups (20–35, 35–50, 50–65 and over 65) over four time periods (1898–1907, 1908–18, 1919–34 and 1935–50) by gender. The four time periods roughly correspond to the periods of change noted earlier. Members under 20 have been excluded due to their small numbers, and the group *over 65* has a small amount of person-years during the first time period. However, as the funds grew bigger, all age groups had a higher number of person-years with time. This measure relates sickness claims to the actual exposure in person-years.^47^

**Fig. 4:**
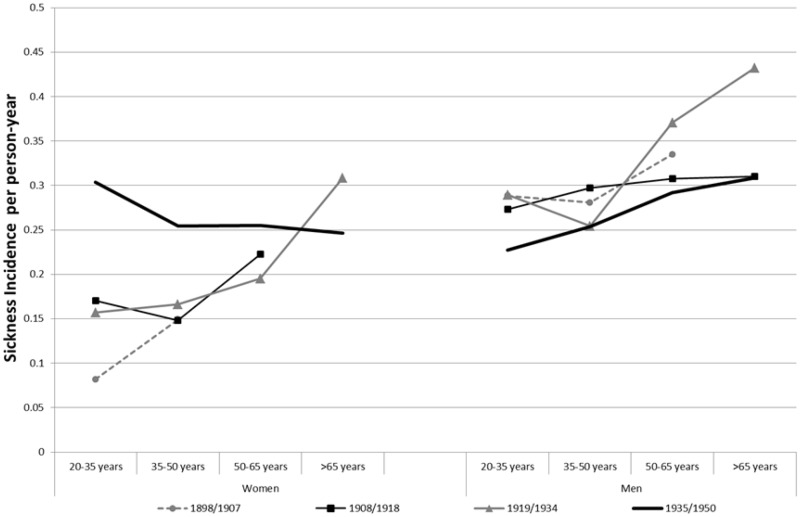
Sickness incidence per person-year in SSBK and Redbar, 1898–1950 *Note*: Data for cases with fewer than 10 sickness cases not shown. *Source*: GSF data set.

The analyses in Figure 4 show that the age distribution of male and female subjects cannot explain the sex differences during the first three periods. Men had a systematically higher number of sickness claims than women in all age groups. Only for the last period were the claims from men and women at the same level, which is consistent with the trend seen in Figure 3.

This graph also shows that there is no evidence of a systematic increase in sickness incidence over time in any age range. There is an increase among women aged 20–35 years between 1919–1934 and 1935–1950, and among men aged over 50 years between 1908–1918 and 1919–1934. Therefore, no systematic age specific increase over time can be seen contradicting the image the aggregated statistics present. However, there is evidence of higher sickness rates for older age ranges, as it has been found by Harris *et al.* The sharpest differences are between the age groups 35–50 and 50–65 years. This indicates that the overall increase in sickness cases for men up until 1933, as seen in Figure 3, was caused mainly by the change in the age composition in the fund: there was a higher proportion of older members with, in general, higher sickness incidence. As membership numbers increased in the period thereafter, more younger members entered the fund. Moreover, the law changes implemented in 1934 also meant that a subsequent sickness case of the same disease that happened within 90 days after the first claim should be counted as the same case, a change that could also help explain the big drop in cases for men after 1933.

Not only the reduced age, but also gender differences after the implementation of the 1931 law, however, were probably related to the funds’ new widened admission policies together with standardised regulations and working procedures. Both SSKB and Redbar required a certificate of good health before accepting new members, but we suspect stricter health screenings in SSKB. The fund’s minutes reveal that long-term sickness was the worst economic fear for the fund. Thus, early detection and treatment was a key measure. There are no indications that SSBK suppressed claims from members, as sickness declarations from doctors were approved without questioning, and members were encouraged to take their illnesses seriously and go to the doctor early so that diseases would not become long-term.^48^ With that, it is possible that the substantial difference between female members in SSBK and in the national statistics can be explained by a stronger health screening.

The strict selection pattern disappeared as a more diversified pool of women entered SSBK from 1934 onwards, also with new types of women, as shown in Table 1. In the earlier periods, the fund was comprised only of seamstresses from a lower social class, who were likely brought up, lived, and worked in unhealthier surroundings compared to the group of women that entered after 1934, which were not only low-skilled workers but also lower professionals. It is possible that they may have had better living conditions, making it less likely for them to become long-term sick but with higher incentives to use the health insurance for short sickness spells. Thus, the GSF data set shows that institutional policies were probably responsible for a heightened version of the gender effects that have been found in the national statistics for Sweden.

**Fig. 5:**
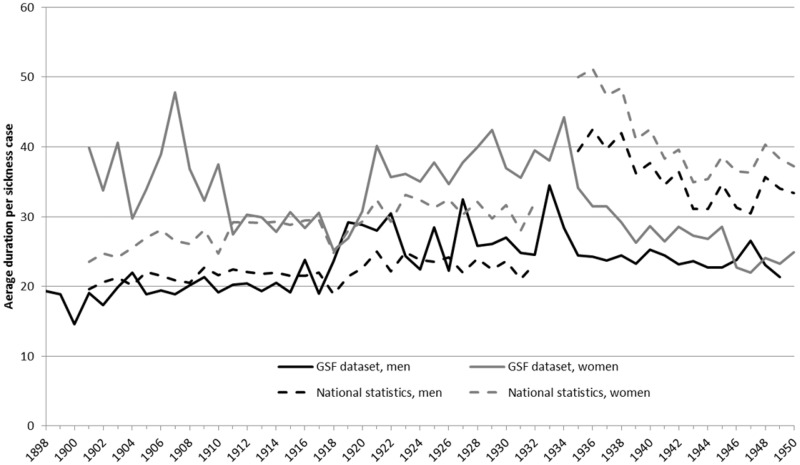
Average duration per sickness case in the national statistics and the GSF data set, 1898–1950. The number of days claimed per sickness case (yearly average). *Source*: GSF data set; Castenbrandt, ‘Trends in Morbidity’.

In contrast to their lower sickness incidence, women’s illnesses were consistently longer than men’s. Meaning that men had more frequent claims but shorter. This is true for both the national statistics and the GSF data set (see Figure 5). Only during the last period do these two groups seem to close in on each other in the GSF data set (while the differences persist in the national statistics), as sickness duration for women decreases.

While male duration remained mostly in line with the national average, the excess duration of female sicknesses in the GSF data set is particularly marked in the first and third periods, which is an effect of changing age composition in the funds, as will be shown later in this section.

After 1934, the national average was considerably higher for both men and women than in the GSF data set. This could be partially explained by the fact that the national statistics also include sickness cases that continued in central funds. With the new law in 1931, all members in sickness funds were automatically part of a central fund and had the right to claim long-term benefits after having used all days at their local fund, leading to longer claims in general. In fact, as the maximum number of days that SSBK and Redbar could provide to its members for a single sickness case was 90 days, the distribution of sickness episodes we have is truncated, and its average, therefore, underestimates the true average.

In order to overcome this problem when analysing sickness duration within the funds, we chose to use the median as a straightforward approach to studying sickness duration trends as it should not be affected by the truncated observations.

When we also take into consideration the age of the members, as we did for sickness incidence, we see that it is not changes over time that is most distinctive; instead, it is the increase with age that is most characteristic. In fact, there is a much stronger relationship between age and sickness duration than between age and sickness incidence. Interestingly, the same has been noticed in Edwards *et al.* research on the Hampshire Friendly Society, where the length of claims rose substantially after 55 years of age.^49^ For both men and women in the GSF data set, the median duration of episodes increased with age, but women experienced longer sickness duration in all ages. The strong effect of the 1931 legislation over sickness incidence cannot be noted for the duration of sickness spells, but the differences in duration remain for women at all ages, so the levels of excess duration we observe seem to correspond to actual gender differences.

However, it is interesting to note two cases of disproportionate increase: when compared with previous periods, women’s sickness duration almost doubled for ages over 50 years in 1919–34, which did not happen for age groups under 35 years. The same thing occurred for men aged over 65 years in 1908–19. While the dramatic increase could reflect the actual impoverishment of the health of older members, we hypothesise that this excess duration could be accounted for by other reasons. We cannot talk about cultural inflation of morbidity because this is a question of duration not of changing thresholds for what is a disease. Also, the need for a medical certificate can be seen as a preventive check for moral hazard, even though cases of unjustly long duration might have slipped through. However, the close social relations in these small communities could explain why members could be more willing to support or make extra allowances for the frailest members, letting them claim longer spells. Especially, as the funds were in good economic conditions during the two periods in question and the relevant sickness cases involved members that had been in them for much of their careers. Thus, it could be another case where fund-specific conditions and regulations heightened differences.

**Fig. 6:**
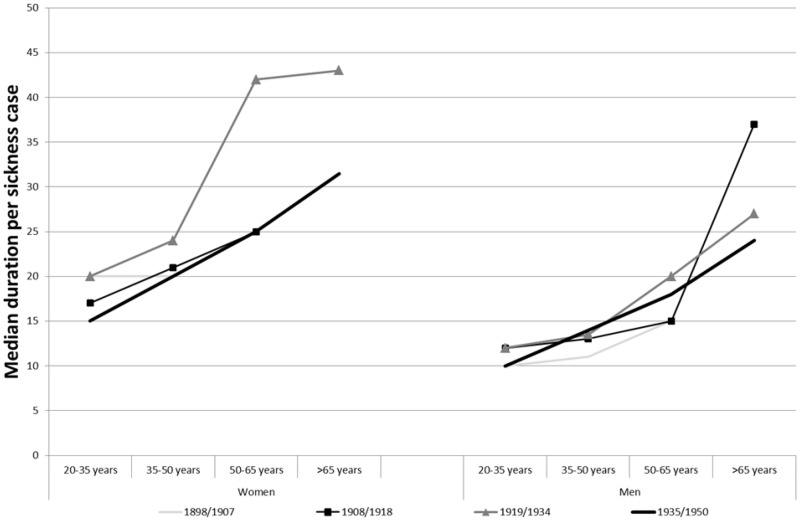
Median duration per sickness case among members in SSBK and Redbar, 1898–1950. Data for cases with fewer than 10 sickness cases not shown. *Source*: GSF data set.

The higher median duration of sickness episodes for women that Figure 6 shows accounts only for a part of the excess sickness duration faced by women. As episodes that lasted more than 90 days were truncated, the median helps us describe the general trend over time. However, it does not properly account for changes in long-term sickness, which were a particular concern at that time. When we look at the proportion of cases that became long term (here defined as lasting 90 days or more), we can capture an additional dimension.

**Fig. 7:**
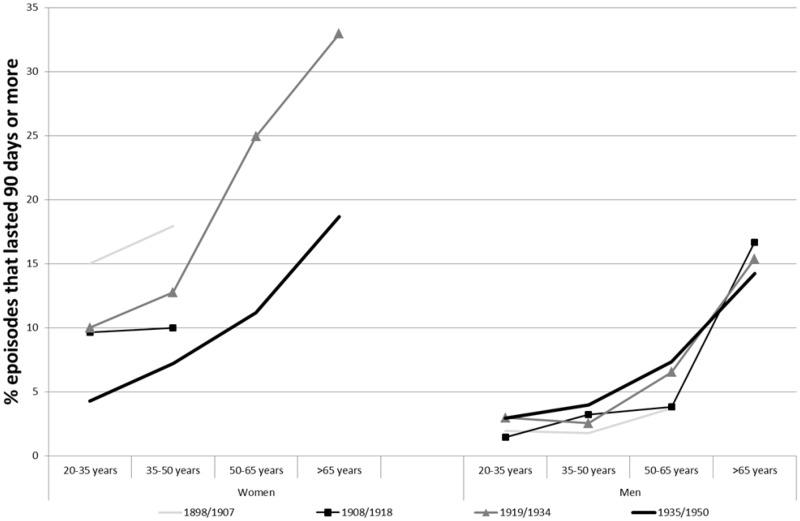
Percentage of sickness episodes in SSBK and Redbar that lasted 90 days or more, 1898–1950. Data for cases with fewer than 10 sickness cases not shown. *Source*: GSF data set.

The overall age structure for the per cent of long-term cases suggest the same situation as the median duration, namely increasing with age (see Figure 7). Moreover, as with median duration, long-term sickness cases were disproportionately experienced by women. For example, in 1898–1907 as many as 15 per cent of all sickness cases among women aged 20–35 years were long-term, while only 2 per cent became long-term for men in the same period and age group.

SSKB’s focus on the dangers that long-term illnesses posed to the survival of the fund suggest that this differential duration was heavily perceived at the time and guided their decisions. In their first years of operation, they addressed this problem and worked towards reducing the number of long-term sickness cases. This happened in several stages. In 1903, the fund decided that members could go to any doctor that they felt most comfortable with and then send the bill to the fund. This was done in order to make sure that members would deal with their illnesses as soon as possible in the hope that this would reduce their duration. Later, in 1912, a doctor was appointed to handle all health certificates for aspiring members. Before this, any doctor could provide such a certificate. The newly assigned doctor was a specialist in chest illnesses and was instructed to look for such health problems so that the fund would not accept new members with illnesses in disease categories that tended to produce the most prolonged cases.^50^ These actions may have influenced the trend towards a reduced proportion of long-term illness among women that we see in Figure 7.

The fact that the proportion of long-term cases decreased from the first to the second period could indicate that the measures taken by SSBK to target this issue had been successful. During the third period, SSBK had a much higher proportion of members over 50. As this group had high numbers of long-term illnesses, this explains the rising number of sick days per sickness claims during this period.

For men, long-term illnesses remained relatively stable. They were, however, influenced by age as cases among members aged over 50 years, and especially over 65 years, lasted longer. For example, in 1935–50, only 4 per cent of members in the age group 35–50 years experienced long-term illnesses, while 14 per cent of cases among members aged over 65 years became long term during that same time.

It is likely that the changes regarding long-term sickness cases made an important contribution to the lower number of sick days per sickness case for women seen in Figure 5 for the second period, 1908–18. By looking at the percentages of long-term illnesses during these periods, it becomes obvious that long-term cases had a more profound influence over women’s sickness cases than over men's (see Figure 7).

In fact, as the relative distribution between age groups changed dramatically, the correlation between increasing number of sick days and increasing age is one of the main explanations behind the rising number of sick days between 1910 and 1934 that is seen for the two funds in Figure 5. The two age groups with members over 50 accounted for only 13 per cent of all members during the time period 1898–1907, while in 1919–34, they accounted for as much as 43 per cent. This rise in the proportion of those over 50 stopped after 1935, at which time the proportion of members between 20 and 35 rose substantially. This helps to explain the stagnant number of sick days after 1935.

## Conclusion

This article has investigated gender differences in sickness claims in the first half of the twentieth century in Gothenburg (Sweden) by creating a database on sickness histories, the GSF data set, which contains information from two sickness funds, Redbar and SSBK, from the period 1898–1950. During their years of operation, the funds transformed from gender-exclusive funds with strict admission regulations to well-integrated parts of a central organisation of sickness funds in Gothenburg, which by 1934 was accessible to everyone from 15 years of age living in Gothenburg. In this process, they went from being small communities, who relied on social control to ensure their survival to larger and more impersonal institutions, which eventually became part of a national health system. Both the changes in fund-specific regulations and legislation had an effect on the access to sickness benefit, which in turn heightened the gender differences.

Overall, we find that there is no evidence of increased morbidity, when we include the changing age-composition of the funds in the analysis. These findings correspond to the conclusions drawn by Harris and Gorsky *et al*. who in their study on the Hampshire Friendly Society found age-specific morbidity to be responsible for the general rise in morbidity as the relative age distribution in the funds changed. While our findings might be specific for SSBK and Redbar, they provide an example that can help us interpret the national statistics.

Even though age composition likely had an effect on the changes after 1934, especially for sickness incidence among men, also other changes such as the extension of the funds, and the more diversified types of women entering the funds, could hold explanatory value. In the earlier period, SSBK comprised only seamstresses from a lower social class, who were likely to have lived and worked under unhealthier surroundings compared to the group of women who entered the funds after 1934, which comprised not only low skilled workers but also lower professionals. It is reasonable to assume that they may have had better living conditions, making them less likely to become long-term sick but perhaps with higher incentives to use their health insurance for shorter spells.

The main contribution of this article is the examination of gender differences. The GSF data set has confirmed the pattern from the national statistics: women had fewer sickness incidences but with a longer duration. In addition, the GSF data set shows the influence of institutional settings, where a strict membership recruitment strategy in SSBK resulted in half the number of sickness cases for women compared to the national statistics.

Gender differences remained when controlling for age composition among members: a higher incidence for men compared to women at all ages. The exception was after 1934, when the new legislation made funds more homogenous, widened their membership recruitment and, thus, loosened admission restrictions to the funds, which raised incidence among women aged 20–35 years to a level higher than men at the same ages. As a consequence, female and male sickness incidence levels converged to the national level. Thus, in terms of sickness cases, we see evidence of the gender differences found for Sweden but heightened by the fund-specific regulations.

Institutional settings, on the other hand, seem to have been less influential over sickness duration as the gender differences were in a similar range to the national statistics. In most periods, the increase in duration gradually increased with age; however, for women in 1919–34, the duration doubled between the age group 35–50 and older ages, and for men in 1908–19, it more than doubled between ages 50–65 and 65+ years. This excess duration for the older ages could be an effect of the close social relations in these small communities with members more willing to support or make extra allowances for the frailest members.

However, the study has shown that the excess duration had another feature. Not only did women have higher median duration of episodes but also the proportion of them becoming long-term was higher. And it was particularly high for older ages, even after the legislation changes implemented in 1934. It would be worth looking into to what extent this is an effect of different disease patterns and occupational groups for men and women. Moreover, there are many external, non-health related, factors related to different gender norms in cultural aspects of disease understanding and reporting, which could help explain differences in sickness absence between women and men.

## Footnotes

1 The name is fictitious to preserve anonymity.

2 See, for example, Martin Gorsky, Bernard Harris and Andrew Hinde, ‘Age, Sickness and Longevity in the Late Nineteenth and Early Twentieth Centuries: Evidence from the Hampshire Friendly Society’, *Social Science History*, 2006, 30, 571–600; Bernard Harris *et al.*, ‘Ageing, Sickness and Health in England and Wales During the Mortality Transition’, *Social History of Medicine*, 2011, 24, 643–65.

3 See, for example, John E. Murray, ‘Social Insurance Claims as Morbidity Estimates: Sickness or Absence?’, *Social History of Medicine*, 2003, 16, 225–45.

4
*Ibid.*; Lars Fredrik Andersson and Liselotte Eriksson, ‘Sickness Absence in Compulsory and Voluntary Health Insurance: The Case of Sweden at the Turn of the Twentieth Century’, *Scandinavian Economic History Review*, 2017, 65, 6–27.

5 Lars-Fredrik Andersson and Liselotte Eriksson, ‘Exclusion of Women and Organisational Characteristics: Swedish Mutual Health Insurance 1901–1910’, *Business History*, 2018.

6 Helene Castenbrandt, ‘Trends in Morbidity: National Statistics on Sickness Claims among the Working Population in Sweden, 1892–1954’, *The Economic History Review*, 2018, 71, 213–35.

7 James C. Riley, *Sick Not Dead: The Health of British Workingmen During the Mortality Decline* (Johns Hopkins University Press, Baltimore, 1997); James C. Riley, ‘Ill Health During the English Mortality Decline: The Friendly Societies’ Experience', *Bulletin of the History of Medicine*, 1987, 61, 563–88; George Alter and James C. Riley, ‘Frailty, Sickness, and Death: Models of Morbidity and Mortality in Historical Populations’, *Population Studies*, 1989, 43, 25–45.

8 John E. Murray, ‘Social Insurance Claims as Morbidity Estimates: Sickness or Absence?’, *Social History of Medicine*, 2003, 16, 225–45.

9 Sheila Ryan Johansson, ‘The Health Transition: The Cultural Inflation of Morbidity During the Decline of Mortality’, *Health Transition Review*, 1991, 1, 39–68; Sheila Ryan Johansson, ‘Measuring the Cultural Inflation of Morbidity During the Decline in Mortality’, *Health Transition Review*, 1992, 2, 78–89.

10 Martin Gorsky *et al.*, “The ‘Cultural Inflation of Morbidity’ During the English Mortality Decline: A New Look,” *Social Science & Medicine*, 2011, 73, 1775–83.

11 Andersson and Eriksson, ‘Sickness Absence’, 24.

12 Castenbrandt, ‘Trends in Morbidity’.

13 Andersson and Eriksson, ‘Exclusion of Women’, 3.

14 Jan Sundin *et al.*, *Svenska folkets hälsa i historiskt perspektiv* (Stockholm: Statens folkhälsoinstitut, 2005), 39.

15 See for example: Andreas Ravndal Kostøl and Kjetil Telle, ‘Sykefraværet i Norge de siste tiårene. Det handler om kvinnene’, *Samfunnsøkonomen*, 2011, 125, 32–42; Nikolay Angelov, *Kvinnors och mäns sjukfrånvaro* (Uppsala: Institutet för arbetsmarknadspolitisk utvärdering (IFAU), 2011), 3–6.

16 Lois M. Verbrugge and Deborah L. Wingard, ‘Sex Differentials in Health and Mortality‘, *Women Health*, 1987, 12, 103–45; Anna Oksuzyan *et al.*, ‘Men: Good Health and High Mortality. Sex Differences in Health and Aging’, *Aging Clinical and Experimental Research*, 2008, 20, 91–102.

17 See for example: Oksuzyan, ‘Men: Good Health and High Mortality’; Will H. Courtenay, ‘Constructions of Masculinity and Their Influence on Men’s Well-Being: A Theory of Gender and Health’, *Social Science & Medicine*, 2000, 50, 1385–401; Arne Mastekaasa, ‘Parenthood, Gender and Sickness Absence’, *Social Science & Medicine*, 2000, 50, 1827–42.

18 Ingemar Åkerlind *et al.*, ‘Sex Differences in Sickness Absence in Relation to Parental Status’, *Scandinavian Journal of Public Health*, 1996, 24, 27–35.

19 Eric Patton and Gary Johns, ‘Women’s Absenteeism in the Popular Press: Evidence for a Gender-Specific Absence Culture’, *Human Relations*, 2007, 60, 1579–612, 1580.

20
*Ibid.*, 1592–603.

21 For Germany, see, for example, John E. Murray, ‘History of Health Insurance in Developed Countries’, in Anthony J. Culyer, ed., *Encyclopedia of Health Economics* (Amsterdam: Elsevier, 2014), 366–70.

22 For a more detailed overview of Swedish legislations regarding sickness funds, see Castenbrandt, ‘Trends in Morbidity’, 217–21.

23 Eva Hesselgren, *Vi äro tusenden**: arbets- och levnadsförhållanden inom svensk textilindustri med särskilt avseende på Gamlestadens fabrikers AB 1890**–**1935* (Stockholm: Almqvist & Wiksell International, 1992) 30.

24 Statistiska centralbyrån, *Historisk statistik för Sverige: Del 1. Befolkning*, 2nd edn. (Stockholm: Beckmans, 1969) .

25 Bertil Andersson, Martin Fritz and Kent Olsson, *Göteborgs historia: näringsliv och samhällsutveckling. 2, Från handelsstad till industristad 1820-1920* (Stockholm: Nerenius & Santérus, 1996), 185–292.

26 K. Kommerskollegii afdelning för arbetsstatistik, *Registrerade sjukkassors verksamhet år 1901*, Arbetsstatistik B (Stockholm: K. L. Beckmans boktryckeri, 1905); K. Pensionsstyrelsen, *Erkända sjukkassor år 1950* (Stockholm: K. L. Beckmans boktryckeri, 1952).

27 Castenbrandt, ‘Trends in Morbidity’, 218–20.

28 See Anders Berge, *Sjukvårdens underklass: Sjukvården i den kommunala fattigvården 1910**–**1950* (Umeå: Borea, 2007).

29 SSBK, vol. 1, Landsarkivet i Göteborg (GLA), Sweden.

30 SSBK, vol. 43, GLA, Sweden.

31 Redbar, vol. 26, GLA, Sweden.

32 Redbar, vol. 4, GLA, Sweden.

33 SSBK, vol. 5, GLA, Sweden.

34 Redbar, vol. 26; SSBK, vol. 43, GLA, Sweden.

35 SSBK, vol. 2, GLA, Sweden.

36 Redbar, vol. 26; SSBK, vol. 43, GLA, Sweden.

37 SSBK, vol. 43, GLA, Sweden.

38 Redbar, vol. 26, §; SSBK, vol. 43, GLA, Sweden.

39 Redbar, vol. 26; SSBK, vol. 43, GLA, Sweden.

40 For Redbar, we have complete coverage of exit dates between 1935 and 1948, additional information from the records for the period 1916–33 and no additional information for the periods 1890–1915 and 1934. For SSBK, the coverage is complete for 1898–1913 and 1934–44; we only have partial information for 1914–15, 1918–30 and 1933 and no additional information for the periods 1916–17 and 1931–32.

41 These are distributed as: 115 = 5.68 per cent of Redbar’s members and 26 = 1.54 per cent of SSBK’s members.

42 Of the 12,197 apparent sickness episodes, we removed 648 because they corresponded to disbursement of money for burial costs or other types of payments (147), or they were, in fact, split into episodes that spanned over 2 years and that we reunited in one (501).

43 Redbar, vol. 26; SSBK, vol. 43, GLA, Sweden.

44 Redbar, vol. 26; SSBK, vol. 43, GLA, Sweden.

45 Bernhard Eriksson, *Sjukkassereformen: Förordning om erkända sjukkassor den 26 Juni 1931, Lag om understödsföreningar den 29 Juni 1912 jämte andra författningar som beröra sjukkasseverksamheten, med inledande översikt och kommentar*, Tidens medborgarhandböcker (Stockholm: Tidens förlag, 1931), 53.

46 Andersson, Fritz and Olsson, *Göteborgs historia*, 27–28.

47 In total, during the funds operating years, the members add up to more than 16 million person-days, equivalent to around 44,000 person-years with exposure to the risk of sickness.

48 Helene Castenbrandt, ‘Administrating Sickness: The Running of a Working Class Women’s Sickness Fund, 1898–1946’, in Axel Hüntelmann and Oliver Falk, eds, *Accounting for Health* (forthcoming).

49 Claudia Edwards *et al.*, ‘Sickness, Insurance and Health: Assessing Trends in Morbidity Through Friendly Society Records’, *Annales de démographie historique*, 2003, 105, 145–47.

50 Castenbrandt, ‘Administrating Sickness’.

## Notes

Helene Castenbrandt is a Researcher at the Department of Economic History at Lund University, Sweden, and an Associate Professor the SAXO Institute at the University of Copenhagen, Denmark. Her research interest lies with questions related to population health and mortality. This research has been financed through the Swedish Research Council.

Barbara Ana Revuelta-Eugercios is an Archivist and Research Associate Professor at the National Archives in Denmark and an Associate Professor at the SAXO Institute at the University of Copenhagen. She is a historical demographer primarily interested in health and mortality studies through the construction of large-scale individual level databases.

Kjell Torén is a Professor of Insurance Medicine at the Section of Occupational and Environmental Medicine, University of Gothenburg, Sweden. His main research area is work disability and occupational risk factors for respiratory and cardiovascular diseases.

